# The Cross Talk between Underlying Mechanisms of Multiple Sclerosis and Epilepsy May Provide New Insights for More Efficient Therapies

**DOI:** 10.3390/ph14101031

**Published:** 2021-10-11

**Authors:** Atefeh Rayatpour, Sahar Farhangi, Ester Verdaguer, Jordi Olloquequi, Jesus Ureña, Carme Auladell, Mohammad Javan

**Affiliations:** 1 Department of Physiology, Faculty of Medical Sciences, Tarbiat Modares University, Tehran 14117-13116, Iran; atefeh_rayatpour@modares.ac.ir (A.R.); s.farhangi@modares.ac.ir (S.F.); 2 Institute for Brain and Cognition, Tarbiat Modares University, Tehran 14117-13116, Iran; 3 Department of Cell Biology, Physiology and Immunology, Biology Faculty, Universitat de Barcelona, 08028 Barcelona, Spain; everdaguer@ub.edu (E.V.); jurena@ub.edu (J.U.); 4 Centre for Biomedical Research of Neurodegenerative Diseases (CIBERNED), Instituto de Salud Carlos III, 28029 Madrid, Spain; 5 Institute of Neuroscience, Universitat de Barcelona, 08035 Barcelona, Spain; 6 Laboratory of Cellular and Molecular Pathology, Biomedical Sciences Institute, Health Sciences Faculty, Universidad Autónoma de Chile, Talca 3460000, Chile; jolloquequig@uautonoma.cl; 7 Cell Science Research Center, Department of Brain and Cognitive Sciences, Royan Institute for Stem Cell Biology and Technology, ACECR, Tehran 14117-13116, Iran

**Keywords:** multiple sclerosis, demyelination, epilepsy, seizure, neurodegeneration

## Abstract

Despite the significant differences in pathological background of neurodegenerative diseases, epileptic seizures are a comorbidity in many disorders such as Huntington disease (HD), Alzheimer’s disease (AD), and multiple sclerosis (MS). Regarding the last one, specifically, it has been shown that the risk of developing epilepsy is three to six times higher in patients with MS compared to the general population. In this context, understanding the pathological processes underlying this connection will allow for the targeting of the common and shared pathological pathways involved in both conditions, which may provide a new avenue in the management of neurological disorders. This review provides an outlook of what is known so far about the bidirectional association between epilepsy and MS.

## 1. Introduction

Multiple sclerosis (MS), the most common cause of non-traumatic neurological disability in young adults, is characterized by inflammatory demyelination in both white and gray matters, followed by axonal injury and loss. Although focal demyelinated lesions are the main hallmark of MS, diffuse inflammation and axonal damage are present in normal appearing white matter (NAWM), as well as in gray matter [1]. Axonal loss is shown to be a feature of clinical disease onset throughout all stages [2]. During the progression of the disease, several structural changes may lead to decreased functional connectivity between neuronal networks, which leads to several complications [3,4]. As a result, patients with MS may develop a wide range of symptoms including paralysis, mental changes, cognitive impairment, depression, and even epilepsy [1].

Epilepsy is one of the most common neurological disorders, involving people of all ages and both sexes, although its prevalence is slightly higher in men [5]. It has higher incidence rates in developed countries, where it can reduce the patient’s life quality [6]. The causes of epilepsy are diverse and involve a variety of pathological mechanisms [7]. People with epilepsy show frequent seizures, severe learning and memory disabilities, cognitive impairments, depression, anxiety, and abnormalities in physical functions [6]. Moreover, epileptic seizures lead to aberrant synchronous discharge in a network of neurons, which might occur after diverse brain insults, such as traumatic brain injury (TBI), stroke, and intracerebral hemorrhages [8,9]. There are three different types of seizures based on the origin within the brain: partial, generalized, and unclassified. Partial seizures initially affect a certain area of the brain. Generalized epilepsy arises when two cerebral hemispheres are affected by abnormal electrical activity and unclassified seizures have an unknown onset [10,11]. The cause of epilepsy in many patients is also unknown, but head trauma, genetic mutations, autoimmune diseases [12], and focal cortical dysplasia [13] are among the possible causes. 

The abnormal synchronous neuronal discharge, a clinical feature of epilepsy, has been reported to be a comorbidity in other many neurological disorders, as well as in neurodegenerative diseases such as Huntington disease (HD), Alzheimer’s disease (AD), vascular dementia, brain tumors, and autism [8,14]. Likewise, epileptic seizures have been accepted as a part of the disease spectrum of MS, and epilepsy is slightly more common in people with MS [15]. In this sense, the association between MS and epileptic seizures does not appear to be a coincidence, but a bidirectional relation. It was not long after Jean Martin Charcot introduced MS as a novel disorder of the central nervous system (CNS), that Wilhelm Leube described an MS patient with seizures in 1871, which was the first evidence of epilepsy in MS [16]. Since then, seizures have been accepted as a part of the disease spectrum in MS. Similarly, although HD, AD, and other neurodegenerative diseases differ in their pathological background and symptoms, epileptic seizures might be involved in their etiology [17,18,19,20]. Indeed, epileptic patients have a higher chance of developing other neurological alterations. Hence, understanding the basis of the link between neurological disorders and epilepsy has important consequences for treatment, diagnosis, and management. 

Even though epilepsy is thought to be a disorder of the gray matter, several experimental and clinical studies have also shown abnormalities in white matter [21,22]. There is growing evidence suggesting that seizures damage the myelin sheaths [23]. Therefore, in the context of MS, seizures can exacerbate demyelination, which reflects the fact that these two diseases have some shared pathophysiology. 

Inflammation is a common pathophysiological mechanism in neurological diseases and epilepsy. In turn, MS involves inflammatory demyelinated lesions in both white and gray matter. Lesions in the spinal cord, brainstem, and cerebellum may lead to involuntary movements in patients with MS [24]. Furthermore, it is suggested that the gray matter lesions and the existing inflammation could trigger a prolonged seizure or a series of seizures occurring in a quick succession that cause epilepsy in patients with MS [25].

The bidirectional connection between MS and epilepsy attracts remarkable attention leading to different hypotheses. Determining whether seizures are an integral part of MS phenotype or are developed secondarily to the progression of MS disease might offer alternative therapeutic approaches. In the following sections, we discuss the prevalence of seizures and other clinical manifestations in demyelinating conditions, and then we provide an outlook of the bidirectional connection between MS and epileptic seizures. Finally, we show a comprehensive and timely summary of the mechanistic pathways governing seizures in MS, as well as myelin damage in the context of epilepsy.

## 2. Seizure Occurrence in the Context of Demyelinating Disorders

Although different demyelinating conditions are associated with seizures, their prevalence and clinical characteristics are different among patients. Epilepsy prevalence is 0.27–1.7% in the general population [26], however, it occurs more frequently in patients with MS [27,28]. In a review of 30 published clinical series representing a total of 19,804 patients with MS, Koch et al. estimated the prevalence of epileptic seizures at the range of 0.5–8.3%, with the average of 2.3% [27]. Similarly, in a large cohort of 5041 patients with MS, the prevalence was 2% [28], which is about three times higher than in the general population. A recent systematic review supported these previous data, estimating that the incidence and prevalence of seizures in patients with MS were 2.28% and 3.09%, respectively [29]. As we mentioned above, in addition to MS, several studies have reported seizures along with other demyelinating disorders, such as antibodies associated demyelinating diseases and progressive multifocal leukoencephalopathy (PML). 

MOG antibody disease (MOGAD) is an inflammatory demyelinating disease of the CNS characterized by the presence of anti-MOG autoantibodies (MOG-Abs). Myelin oligodendrocyte glycoprotein (MOG) is a membrane protein expressed on the outermost surface of myelin sheaths, which is thought to be important in the myelination process. MOG-Abs have been strongly associated with several demyelinated disorders such as acute disseminated encephalomyelitis (ADEM), pediatric MS, transverse myelitis, optic neuritis (ON), and neuromyelitis optical spectrum disorders (NMOSD), but are rarely detected in patients with MS [30,31,32]. There is growing evidence reporting a link between the presence of MOG-Abs and seizures, occurring in combination with demyelination or even as an isolated phenomenon [33,34,35,36]. In several case reports, patients with MOG-Abs developed seizures as the first sign prior to demyelination or in a subsequent disease course [34,36,37]. However, the clinical spectrum of these seizures and the contribution and importance of MOG-Abs on seizure development remain unclear. In addition to MOG-Abs, antibodies against AQP4 (water channel protein aquaporin-4) have also been considered as a sensitive and highly specific serum hallmark of the NMOSD [38]. AQP4 is a protein required for a normal rate of water exchange across the blood–brain interface. Consequently, in 2015, the International Panel for antigen NMO Diagnosis (IPND) revised the diagnosis criteria based on the presence or absence of AQP4-Abs [39]. Moreover, a proportion of patients who met the criteria for NMOSD but lacked AQP4-Abs were seropositive for MOG-Abs [38,39]. Both associated demyelinating diseases develop seizures, however, they are more common in patients with MOG antibody-associated demyelination than in patients with AQP4 antibody-associated demyelination [33], which is thought to be related to cortical and subcortical lesions [40]. Even though seizure occurrence was highly associated with gray matter lesions, the prevalence of seizures was 18% in patients with progressive multifocal leukoencephalopathy (PML), in which demyelination is thought to be restricted to the white matter [41]. More details on the characteristics of patients with demyelinating disorders who developed seizures are summarized in Table 1.

### 2.1. Seizures as A Clinical Manifestation of Multiple Sclerosis (MS)

Several studies have reported that patients with MS are three to six times more likely to suffer from epileptic seizures than the rest of the population [51]. However, the origin, the extent, and the importance of epileptic seizures in patients with MS remain ambiguous. Some studies have suggested that seizures could affect patients at any stage of the disease’s progression. It is also suggested that the severity and course of MS might be correlated to the occurrence of epilepsy [52]. In this sense, a recent retrospective cohort study with 14,545 MS cases and 43,635 controls have strongly suggested a direct association between the severity and duration of MS and the incidence of seizures. While the cumulative incidence of epilepsy in relapsing-remitting MS (RR-MS) was 2.2%, this value in patients in progressive stage was 5.5%, and continuously increased to 5.9% with increasing the duration of disease in patients with disease duration of ≥34 years. Furthermore, patients whose Expanded Disability Status Scale (EDSS) score was more than 7 had a cumulative incidence epilepsy of 5.3% [52]. Although patients with progressive forms of the disease might be more likely to suffer from epilepsy [52,53], some researchers suggested that seizure might be the first clinical manifestation at the time of MS diagnosis in 10.5% of patients [28,42]. Based on radiological findings, Hussona and co-workers reported a case series of patients whose only clinical manifestation were seizures, and their radiological findings showed abnormalities compatible with demyelination, which meets the criteria for clinically isolated syndrome (CIS) or early MS [54]. In addition, it has been shown that seizures may occur during the relapse in a subset of patients [42], which could be associated with cognitive impairment. Furthermore, seizures are more common in patients with early-onset MS (5.5% in pediatric MS) [26,55], which have poor prognosis toward disability and death. In turn, decreased brain volumes and poor cognitive function are also reported in patients with frequent or uncontrolled seizures [26]. Interestingly, patients with seizures appearing at MS onset or relapses usually do not experience recurrent seizure; by contrast, those patients with seizures associated to cognitive impairment and progression of the disability were more likely to experience recurrent seizure [42]. Additionally, a study developed in a cohort of 5041 patients with MS has suggested that there are no differences in gender, duration, and course of MS between those patients with MS suffering from seizures and those who never experienced seizure in any course of their disease [28]. This notion is also supported by other studies [26,45].

The fact that almost all type of seizures have been associated with MS [27] suggests an involvement of MS pathological mechanisms in the etiology of seizures, even though partial seizures with secondary generalization have been shown to be the most prevalent in patients with MS [42], and a review of 30 studies have shown a similar prevalence of primary or secondary-generalized seizures, which accounts for nearly two-thirds of all seizures in MS [27]. Electroencephalography (EEG) analyses in patients with MS reported abnormalities in brain electrical activity, such as asynchronous theta activity, synchronous rhythmic slow waves, and focal flattened EEG patterns [42]. Moreover, in a cohort of 5041 patients with MS, 63% of those who experienced seizures also showed abnormal EEG. Importantly, slow background, focal spikes, focal waves, and ictal discharge have also been reported in patients with MS (Table 2) [28]. In spite of this, the incidence of seizures in patients with MS does not follow a similar pattern. Hence, in some cases, seizures occur rarely and are associated with relapses, while in others, seizures may be the first signs of MS disease at the time of diagnosis. In addition, some patients with MS with cognitive impairment suffer from seizures recurrence [26,28,42,55]. 

Since epilepsy is a significant comorbidity in MS, clarifying whether the patients with MS are at increased risk for developing seizures is important. Moreover, it is unclear to what extent seizures can exacerbate the clinical course and long-term prognosis of MS.

### 2.2. Possible Pathophysiological Processes Underlying Seizure Development in Patients with MS

The pathophysiological mechanisms that explain the link between MS and epilepsy are still under investigation. Although MS was initially conceptualized as a white matter demyelinating disease, it is now well known that gray matter lesions (GMLs) and atrophy are more frequent than previously suggested [57]. In addition, there is evidence that gray matter lesions occur from the earliest stages of the disease [58]. This leads to the hypothesis that cortical lesions in MS may play an epileptogenic role, explaining the seizures’ appearance in patients with MS. This notion is well supported by studies based on the magnetic resonance imaging (MRI), longitudinal studies, and postmortem tissue analyses that have shown more cortical lesions and atrophy in patients with a higher prevalence of epilepsy [26,59]. Furthermore, some MRI and double inversion recovery (DIR) repetitive studies have revealed that formation of new juxta-cortical and/or cortical lesions are associated with generalized seizures in most patients with MS with epilepsy [25,44,60], suggesting an involvement of cortical and subcortical lesions in the development of seizures. Although it appears to be an association between the extent of cortical and subcortical lesions and the presence of seizures, not all patients with MS with gray matter lesions developed seizures [45]. Therefore, it is likely that lesions in some cortical regions are more prone to trigger seizuregenesis. In this regard, Calabrese and co-workers have reported severe damage in the temporal lobe of RR-MS, with seizures compared to those patients without seizures. Regional analysis revealed that the most affected gray matter regions in RR-MS epileptic patients were the hippocampus (14.2%), the lateral temporal lobe (13.5%), the cingulate (10.0%), and the insula (8.4%). Furthermore, cortical thinning was observed in the middle temporal gyrus, fusiform gyrus, cingulate gyrus, and in the insula of epileptic RR-MS, compared to other patients with RR-MS [45]. Collectively, the gray matter atrophy and neuronal loss in patients with MS appear to occur in structures associated with mesial temporal lobe epilepsy. In another study, Nicholas et al. confirmed middle temporal gyrus thinning and the loss of GABAergic interneuron in layers IV and VI by examining the postmortem entorhinal cortex of patients with MS with seizures. Even though the loss of inhibitory interneurons seemed to be related to GMLs, it was not explained by inflammation and mitochondrial dysfunction within the type I gray matter lesions [46]. 

Abnormalities in the GABAergic system may be associated to seizure incidence in patients with MS. In this sense, a recent study has revealed a selective vulnerability of inhibitory interneurons to demyelination. There is a specific loss of parvalbumin-positive GABAergic interneurons in the cortex of postmortem secondary progressive MS (SP-MS), suggesting that specific interneuron subtypes are vulnerable to neurodegeneration in the cortex of patients with MS. In an animal model of cortical demyelination, it has also been confirmed that the selective susceptibility of parvalbumin fast spiking interneurons are secondary to cortical demyelination [61]. Since the balance between excitatory and inhibitory activities is crucial for the maintenance of the neuronal network’s stability, a reduction in inhibitory neurons may trigger epilepsy in patients with MS. The loss of inhibitory interneurons is exacerbated by disease progression, supporting association between the severity and duration of the MS, and the incidence of seizures [52]. In turn, by using magnetic resonance spectroscopy (MRS), Cao and co-workers also reported abnormalities in the GABAergic system. Hence, the level of GABA concentration was lower in the posterior cingulate cortex and the left hippocampus of RR-MS, which was likely to be due to GABAergic neuronal loss [47]. 

In addition, a reduction in ATP production in demyelinated lesions and disturbances in ion homeostasis may induce Ca^2+^-mediated degeneration in GABAergic inhibitory interneurons in the MS motor cortex [43]. Given that hippocampus is more susceptible to energy failure mediated by mitochondrial dysfunction [62], impaired ATP production as a consequence of inflammatory demyelination may lead to inhibitory interneuron degeneration, leading to disturbances in the excitatory-inhibitory balance of the neuronal network. Another possible mechanism underlying hyper-excitability following demyelination is a switch in sodium channel expression within the neurons whose axons have been damaged. This abnormal sodium channel expression may activate silent sodium channels, leading to hyper-excitability and abnormal impulse activity. This additional mechanism may contribute to the pathophysiology of epileptic seizures in patients with MS [63].

Taken together, epileptic events in patients with MS might be a consequence of gray matter atrophy, hippocampal lesions, and GABAergic interneuron loss. However, there are few studies focusing on the molecular mechanisms, which trigger seizures in MS. Additionally, available data on the mechanisms of neurodegeneration and the mechanisms by which inhibitory interneurons are more vulnerable to degeneration are insufficient. Even though cortical lesions are found in most of patients with MS with seizures, it remains unclear whether specific neuronal networks are more vulnerable to demyelination and undergo degeneration. If cortical lesion load increases the risk of epilepsy, as suggested by several researchers, an increased prevalence of epilepsy among patients with SP-MS should be expected. However, there are still controversies regarding which clinical characteristics of MS are associated with the occurrence of epileptic seizures. 

While the demyelination-induced seizure has been addressed for decades in patients with MS, there are still two open questions without a definite conclusion: (1) How might myelin deficiency lead to neuronal hyper-excitability? (2) What are the contributions of glial cells? Given that the notion that epilepsy occurs due to changes in neuronal properties has now been challenged [64], which cellular and molecular changes during demyelination may lead to alterations in neuronal activity triggering seizuregenesis? To answer this question, the use of cuprizone as an experimental model of demyelination has reported new data in this field.

### 2.3. Cuprizone Induced Demyelination as a Model for Epilepsy

Since its first application in research, cuprizone (CPZ) has been used to study the processes involved in de- and remyelination, despite the fact that its exact mechanisms of action remain rather elusive. Some preliminary studies have reported the occurrence of seizures in mice fed with 0.3% CPZ diet for 7 weeks; however, the mechanisms underlying the seizures were not addressed in these studies [65,66]. Indeed, research in this area did not characterized seizures induced by CPZ until 2008, when Hoffman et al. reported that chronic CPZ diet treatment at the same concentration used for induction of MS model led to short but frequent spike neuron discharges in the EEG. In addition, cuprizone-treated mice exhibited generalized tonic clonic seizures induced by handling or other types of sensory stimulation. Furthermore, a massive demyelination and axonal degeneration were reported in the dorsal and ventral areas of hippocampus formation, areas often involved in seizures [67]. However, the role of glial cells and the susceptibility of different neuronal subpopulations remained unknown. In this sense, a recent study has revealed that chronic demyelination induced by CPZ leads to seizure activity in the dorsal hippocampus. Furthermore, some changes were found within the CA1 pyramidal hippocampal area after 9 weeks of CPZ treatment, including extensive demyelination, loss of parvalbumin (PV^+^) inhibitory interneurons, widespread gliosis, and a transient decrease in aquaporin-4 (AQP4) expression [68]. Loss of interneurons following CPZ feeding is not surprising, since half of the cortical myelin located in layer 2/3 and a quarter in layer 4 ensheathes the axons of inhibitory neurons, especially parvalbumin-positive basket cells [69]. Reactive astrogliosis is a common feature of demyelination and seizure, which is found in both human postmortem and animal models [64,70]. In this cuprizone model, an increase of Kir4.1 protein was revealed.

Glial cells have a significant role in modulating brain water transport through AQP4 channels [71]. Therefore, changes in its expression, as occurs in the cuprizone experimental model, may disturb osmolality and the resting membrane potential and subsequently lead to hyper-excitability [72]. AQP4 downregulation was also reported in seizure animal models [73]. Furthermore, AQP4-lacking mice have a prolonged seizure duration, which confirms its role in the maintenance of homeostasis [72]. Since AQP4 is indirectly involved in potassium homeostasis, changes in the expression of inward rectifier potassium channel Kir4.1 might be involved in seizuregenesis by increasing the extracellular potassium concentration [74].

A complete understanding of the pathological mechanisms underlying CPZ intoxication could be of the greatest importance to study the seizure development secondary to demyelination and to claim the validity of MS and epilepsy therapies developed in this model. Further studies are needed to elucidate the effects of CPZ on different hippocampal interneuron subtypes and their role in the initiation of seizures. Likewise, a deep understanding of the pathophysiology underlying the inhibitory cell loss in chronic demyelination may pave the way for a better understanding of seizure development secondary to MS and its clinical management.

### 2.4. Common Inflammatory Molecules/Pathways Underlying Demyelination and Seizuregenesis: Role of Glial Cells

As we have already explained, despite the different etiology underlying MS and epilepsy, inflammation is a common feature in their pathophysiology [75]. Although the ultimate cause of MS is yet to be clarified, it is widely accepted that autoimmunity plays a key role in its pathophysiology. Hence, it has been proposed that peripheral T-lymphocytes enter the CNS, triggering the initial lesions [76]. Specifically, autoreactive CD8^+^ and CD4^+^ T cells seem to activate microglial cells, initiating inflammatory-induced lesions in the initial phases of the disease [77]. In this sense, despite the specific autoimmune reaction in MS still being uncharacterized, the axonal pathology co-localizes with tissue-damaging CD8^+^ lymphocytes and over-activated CD4^+^ T-cells [78]. Moreover, immunomodulatory treatments reduce disease’s relapses and disability progression, which, in turn, suggests a key role for inflammation [79]. Indeed, it has also been proposed that the inflammatory response in MS might occur secondarily to a primary infection or a neuronal disturbance [80]. In any case, although other mechanisms such as altered iron homeostasis, oxidative stress, and mitochondrial injury have been involved in MS [81], a close association between inflammation and neurodegeneration in all lesions and disease stages is clear [81,82]. Alterations in the inhibitory–excitatory balance, which could result from lesions and structural changes, may also lead to epileptic seizures. Indeed, structural changes such as neuronal loss, astrogliosis, and microgliosis are common epileptogenic factors, which also happen during demyelination. Astrocytes react to almost all types of pathological alterations in CNS homeostasis by significant morphological and molecular changes termed astrogliosis, which include proliferation, hypertrophy, and functional changes. Astrogliosis can be triggered by various signals from dying cells, hypoxia, and reactive oxygen species (ROS), including nitric oxide (NO). Moreover, innate immunity mediators such as IL-1β, tumor necrosis factor-α (TNF-α), and lipopolysaccharide (LPS) could affect astrocytes activity, as reviewed in detail elsewhere [83]. In turn, astrocytes modulate immune responses by releasing cytokines such as interleukin (IL)-8 or CXC chemokine receptor-8 (CXCL8), IL-6, TNF-α, and IL-1β [84]. They can also control microglial activation [85] and express various immune-associated receptors, such as mannose receptor, Toll-like receptors (TLRs), NOD-like receptors (NLR), and components of the complement system [86]. Hence, since glial cells play a very prominent role in regulating the homeostasis of neuronal microenvironment and synaptic transmission [87], switching from normal to reactive glia due to inflammatory demyelination, is sufficient to cause changes in some neuronal properties. In this sense, several inflammatory mediators such as HMGB1, IL-1B, IL-6, TNFα, and different chemokines have been shown to change neuronal properties and lead to hyper-excitability [88,89,90,91]. Common inflammatory molecules/pathways underlying seizuregenesis and demyelination are summarized in Table 3.

Reactive astrocytes also secrete extracellular matrix (ECM) components that participate in MS pathology. Since changes in ECM molecules are highly suggested to contribute to neuronal cation currents [92,93], it seems that reactive astrocytes fail to regulate the neuronal milieu within the demyelinated lesions. Consequently, gliosis and inflammation can potentially lead to seizures in patients with MS. 

**Figure 1:**
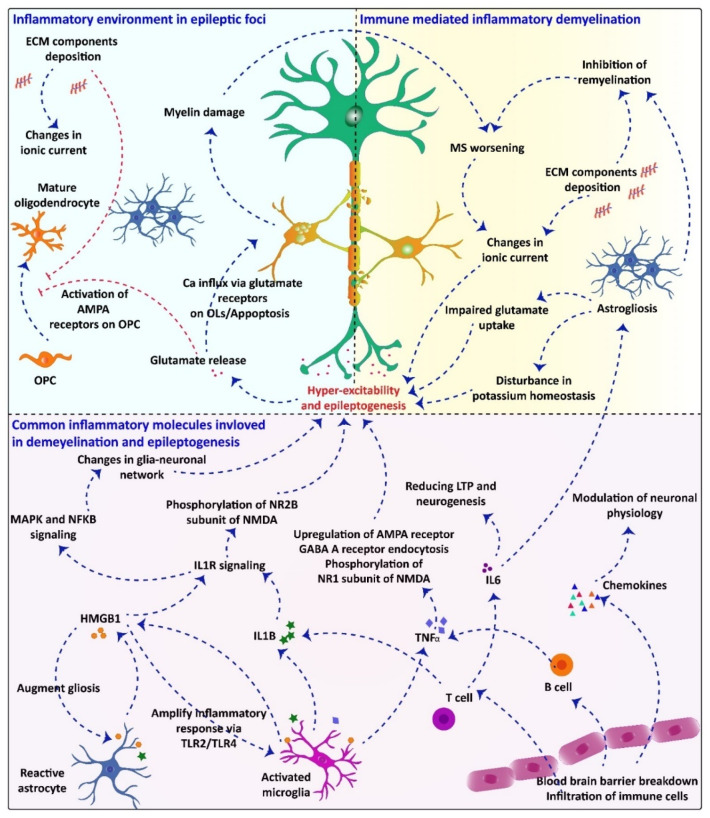
The common inflammatory mechanism underlying demyelination and epilepsy and how one can pathology accelerate the other is proposed: Inflammatory processes in MS pathology are also involved in the etiology of seizures and trigger hyper-excitability. In turn, inflammation, gliosis, ECM component deposition, blood–brain barrier breakdown, immune cells infiltration, and production of inflammatory mediators such as IL-1B, IL-6, HMGB1, TNF-α, and chemokines might stabilize a common mechanism in the pathophysiology of seizures and epilepsy in MS. Phosphorylation of NR2 subunit of NMDA receptor by IL-1B and HMGB1 may lead to an enhanced NMDA activity and Ca^2+^ influx into neurons. Different types of cytokines are released by infiltrated or resident immune cells within the demyelinated lesions. These cytokines can modulate neuronal physiology by changing voltage-gated channels and enhancing discharge of some neurotransmitters. Furthermore, pro-inflammatory cytokines and IL-1B inhibit astrocytic glutamate uptake, leading to hyper-excitability and, subsequently, seizures in the context of MS. Releasing high amount of glutamate and impaired glutamate uptake can induce apoptosis in oligodendrocytes via influx of large amount of Ca^2+^ through glutamate receptors. This process exacerbates myelin damage and worsens MS.

Similarly, although the primary insult leading to an active epileptic condition is unknown in many patients, studies performed in animals with either acquired or genetic epilepsy have unveiled some alterations in neurons, glia, and blood vessels [94]. Thus, it has been shown that voltage-gated and receptor-gated ion channels are altered by transcriptional and epigenetic mechanisms during the development of epilepsy (epileptogenesis process), leading to neuronal hyper-excitability [95,96,97]. In turn, the increased neuronal activity triggers an increased CNS innate immune response, leading to production and release of inflammatory mediators and oxidative stress [12]. While peripheral macrophages infiltrate brain parenchyma, there is an activation of microglia that feeds the neuroinflammatory and hyper-excitability processes [98,99], and could promote a dysfunction of the blood–brain barrier (BBB) [100,101]. All these changes are involved in the onset and progression of epilepsy, cell death and neurological comorbidities. Bearing in mind the above-mentioned, inflammation is being recognized as the common feature in the pathophysiology of MS and epilepsy [75,102] (Figure 1). 

Collectively, the overall pathology of MS and epilepsy seems different, but some shared mechanisms are undoubtedly present. Each of these two diseases may lead to the occurrence of underlying mechanisms of the other. Therefore, a positive feedback could exist, contributing to the worsening of these conditions. The inflammatory environment of MS lesions modulates neuronal physiology and increases the neuronal excitability, which, in turn, may lead to the development of epileptic seizures. Glutamate released during discharges has several effects on oligodendrocyte lineage cells. This process finally leads to myelin damage that exacerbates MS. The common pathways underlying the pathology of both diseases, how MS pathology can trigger epileptic seizures, and how epilepsy leads to myelin damage and subsequently worsens MS, are proposed in Figure 1. Notwithstanding, further preclinical and clinical studies are required to reveal the precise molecular mechanisms involved.

**Table 3:** Common inflammatory molecules/pathways underlying seizuregenesis and demyelination.

Molecules/Receptor/Pathway	Source	Role in Demyelination	Role in Seizuregenesis	Ref.
HMGB1	Astrocytes Microglia Neurons	Microglial proinflammatory response by production of proinflammatory factors (TNF-α, nitric oxide, interleukin-1b (IL-1b), IL-6, CXCL10 and CCL2	Microglial activation via the TLR4/NF-κB signaling pathway Activation of IL-1R/TLR signaling in neurons Phosphorylation of the NR2B subunit of NMDA receptor, which leads to enhanced NMDA activity and Ca^2+^ influx into the neurons	[88,103,104]
TGF-β	Microglia	Induces astrogliosis Promotes Th17 cell differentiation	Astrocyte activation Inflammation Reduced inhibitory transmission	[105,106,107]
TLRs	Microglia	Production of IL-1, IL-6, and IL-12 Induce differentiation of naïve T cells into Th1 and Th17 cells	Hippocampal hyper-excitation via upregulating proinflammatory cytokines such as IFN-β in microglia and astrocytes Up regulation of proinflammatory cytokine such as IL1B, TNF-α, IL-6	[91,108,109]
Hyaluronan/CD44	Astrocytes/ Microglia	Activation of NFƙB to produce pro-IL1B and other proinflammatory cytokines Inhibition of OPC maturation	Hyaluronan plays a permissive role in MFS	[110,111,112]
mTOR	Astrocytes Microglia	Microglial proinflammatory activation Regulation of adaptive and innate immune response T cell proliferation	mTOR pathway regulation depends on glutamate receptor activation A strong link between neuronal hyper-excitability and aberrant mTOR activation	[113,114,115,116]
IL-1B	Macrophages T cells	Proinflammatory response through activation of IL-1R and NF-κB pathway BBB breakdown	Activation of neuronal sphingo myelinaseand Src kinases Phosphorylation of the NR2B subunit of NMDA receptor Reduction in GABA-mediated inhibition of glutamate uptake by astrocytes	[90,117,118,119,120]
TNF-α	Macrophages T cells B cells Neurons	Mediate apoptosis and chronic inflammation through TNFR 1	Inhibition of glutamate uptake by astrocyte Up-regulation of AMPA receptors Phosphorylation of the NR1 subunit of the NMDA receptor Induction of GABA A receptor endocytosis	[91,106,117,121,122]

HMGB1: High mobility group box 1; TLRs: Toll-like receptors; CXCL10: C-X-C motif chemokine 10; CCL2: chemokine C-C motif ligand 2; MFS: Mossy fiber sprouting; NMDA: N-methyl-d-aspartate; AMPA: aminomethyl propionic acid, mTOR: Mammalian target of rapamycin.

## 3. White Matter Abnormalities following Epileptic Seizures

Gray matter, which includes cell bodies of neurons, is markedly damaged in epilepsy [10,123]. Although epilepsy is considered as a gray matter disorder, many experimental and clinical studies have also revealed abnormalities in white matter. In this section, we focus on myelin/white matter changes in animal experiments and patients with epilepsy. We will finally evaluate the pathophysiological mechanisms involved in this abnormality.

### 3.1. White Matter Disorders in Animal Models of Epilepsy

Experimental animal models of epilepsy have been widely used to clarify epileptic mechanisms and to improve the therapeutic strategies of this disorder [124]. For instance, spontaneous recurrent seizures can be triggered in rodents by injection of chemoconvulsants, which include pilocarpine (a muscarinic acetylcholine receptor agonist) [125] and kainic acid (an L-glutamate analog) [126]. Animal studies have revealed that the hippocampus is often involved and obviously damaged in epilepsy. Thus, a study developed in the lithium–pilocarpine rat model displayed white matter changes in this part of the brain, such as a significant reduction in myelin sheaths, myelin basic protein (MBP) expression, myelin thickness, and the volume of myelinated fibers [127]. However, there is a difference in hippocampal MBP expression between immature and adult animals treated with lithium–pilocarpine. Hence, MBP protein is downregulated in both; however, in immature animals, MBP protein gradually increases without reaching normal levels, while in adults, its expression gradually decreases [128]. In addition, 10 weeks after induction of neocortical focal epilepsy, myelin damage and astrocyte/microglia activation was observed in the hippocampus [129]. Furthermore, in the pentylenetetrazol (PTZ; a GABA_A_ receptor antagonist) model of epilepsy, demyelination of the hippocampus in both early and chronic stages has been reported [130]. Moreover, a vast majority of demyelination in the hippocampal regions was also seen in the electrical kindling-induced epilepsy model, which is produced by focal electrical stimulation of the brain [131]. 

There is growing evidence of myelin damage and white matter abnormalities of other brain regions in animal models of epilepsy. Therefore, it seems that the hippocampus is not the only region affected after aberrant neuronal discharge. For instance, the density of myelinated axons in optic tracts and stria medularis appears to be decreased in the kainic acid model [132]. Furthermore, delayed myelination is observed in the brain stem, cerebellum, and cerebral hemispheres in seizure-prone FAST rats on postnatal days 5 and 11 [133]. The expression of MBP protein in the thalamus is also altered in animals with genetic absence epilepsy [134]. Additionally, corpus callosum (CC), the largest forebrain commissure, also appears to be damaged in experimental models of epilepsy [135,136,137]. Given the essential role of CC on transferring information between the hemispheres, epileptic activities can easily be present in the whole brain [138]; however, white matter alterations can occur prior to epileptic seizures and neuronal damage. Indeed, there is evidence of hypo-myelination in the brainstem’s myelinated tracts and cerebellum white matter much prior to the occurrence of epileptic seizures in the spontaneously epileptic rat model [139]. Besides, in some parts of the brain, such as the cortex, thalamus, and basal ganglia, myelin is damaged before neuronal degeneration in the kainic acid model of seizures [140]. 

### 3.2. White Matter Alterations in Patients with Epilepsy

Beyond animal experiments, myelin and white matter integrity have been examined in patients with epilepsy. Therefore, in this section, we provide an overview of studies on white matter changes in human studies. 

Different methods which can be efficiently used in the evaluation of white matter and myelin in epilepsy were recently reviewed by Drenthen et al. [23]. Diffusion tensor imaging (DTI) is a noninvasive method to evaluate white matter integrity by measuring water diffusion along white matter tracts and its directionality in three dimensions. Fractional anisotropy (FA) and mean diffusivity (MD) are the most important parameters used in this technique to measure the white matter integrity and to localize white matter lesions. It has been demonstrated that tightly-packed white matter tracts show highly directional diffusion (high FA and low MD), whereas lesioned tracts display a decreased directional diffusion (low FA and high MD) [141]. DTI has also been used to evaluate white matter abnormalities in epileptic patients. In this regard, studies in patients with temporal lobe epilepsy (TLE), the most common form of focal epilepsy in humans, have shown decreased FA and increased MD in the hippocampus ipsilateral to seizure focus [142,143]. Additionally, DTI analyses have demonstrated a reduced FA in normal-appearing white matter surrounding the lesion in patients with partial intractable epilepsy [144]. Furthermore, lower FA has been reported in two major frontotemporal white matter tracts, including uncinate fasciculus (UF) and arcuate fasciculus (AF) on the side of seizure onset [145]. 

Abnormalities in white matter have also been evaluated by quantitative MRI studies, which have shown a reduction in the volume of temporal lobe white matter ipsilateral to the focal onset in TLE patients [146]. Although the disruption of white matter integrity is more severe in the ipsilateral hemisphere, the contralateral hemisphere could be involved as well [147,148]. Moreover, there are some differences in white matter volume changes between patients with right TLE and those with left TLE. Hence, the volume of CC is significantly decreased in both groups of patients; however, the volume of the prefrontal cortex and fornix is decreased in individuals with left TLE and those with right TLE, respectively [149]. While the patients with right TLE exhibit mostly ipsilateral white matter changes, the patients who experience left TLE display bilateral widespread white matter abnormalities [150], suggesting that white matter abnormalities in patients who suffer from left TLE are more severe than those with right TLE [151]. The widespread white matter abnormalities can be persistent even one year after seizure foci resection [152]. 

Besides the animal studies confirming the CC damage, this structure is also reported to be affected in epileptic patients [22,153,154,155]. Hence, quantitative MRI studies have shown a reduction in the volume of CC [156,157]; however, the results in this regard are controversial. Using the MRI technique, Kim et al. have shown focal lesions in the splenium of CC, although they believe that this abnormality is the result of the toxicity of antiepileptic drugs and could be reversible [158]. Conversely, Oster et al. demonstrated that focal lesions in this area are associated with seizures, rather to than a consequence of antiepileptic drugs [159].

Although it has been suggested that the volume of CC is reduced in patients with epilepsy, this abnormality was not associated with the onset age of the disease [160], and the severe volume reduction of CC at childhood-onset epilepsy compared to the late onset of the disease has challenged this concept [161]. Since the posterior part of CC appears to be more vulnerable to damage at the early onset of seizures, white matter damage seems to be associated with the age of seizure onset [162,163]. In addition, DTI analyses of patients with focal TLE display a reduced FA in the posterior CC, with a significant positive correlation between diffusion anisotropy and the onset age of the seizure [164]. Whereas patients with TLE (left or right) display a reduced thickness of posterior CC, the early onset of left TLE additionally decreases the thickness of both the anterior and midbody of CC [165]. Hence, an early seizure onset seems to be an important factor in the severity of CC damage. 

In addition to CC, other brain structures could also be affected in individuals with childhood-onset seizures [166,167]. In this regard, Hermann and co-workers have demonstrated that white matter damages were not limited to the temporal lobe of TLE patients with childhood-onset; instead, they were associated with total white matter reduction in both ipsilateral and contralateral hemispheres. Similarly, earlier onset of epilepsy causes poorer cognitive abilities and performance when compared to patients who were afflicted later [168]. Collectively, these results indicate that early-onset epilepsy is associated with more severe white matter damage and cognitive impairments.

### 3.3. Pathophysiological Mechanisms Underlying White Matter Disruption in Epilepsy

As has been explained above, there is mounting evidence regarding white matter abnormalities in epilepsy; however, the exact underlying mechanisms remain elusive. It has been suggested that the microenvironment of epileptic foci influences oligodendrocytes progenitor cells’ (OPCs) maturation and, subsequently, the myelination process. In this regard, studies on patients with intractable epilepsy have shown that the proliferation of OPCs increases in the epileptic foci; however, they were unable to differentiate to mature oligodendrocytes (OLs) [169], which might affect the normal function of neurons. This notion was also observed in the acute and chronic phases of the lithium–pilocarpine model [170]. Furthermore, neurons with epileptiform discharge release more glutamic acid, which inhibits the differentiation of OPCs through their interaction with AMPA receptors on the surface of OPCs [171].

BBB disruption, which is an important characteristic of epileptic seizures, might lead to the entrance of peripheral anti-MBP antibodies to the white matter and damage the myelin in the PTZ model of epilepsy, contributing to autoimmune response [130]. In addition to the autoimmune abnormality, OLs’ destruction can occur in demyelination-associated epilepsy. Epileptic seizures trigger pathophysiological changes resulting in oxidative stress and mitochondrial injury [172]. Furthermore, experimental and clinical studies have demonstrated that seizures lead to inflammatory responses affecting the permeability of BBB, as well as to an immune cell infiltration into the CNS [173]. OLs are the most susceptible cells to injury among other cellular components of the CNS. Therefore, exposure to pathological conditions, as described above, can obviously destroy OLs and their myelin sheaths [174]. 

In addition, numerous studies have shown that excitotoxicity, one of the most important mechanisms of epilepsy due to the increased levels of glutamate in synapses, can also damage OLs [175]. Indeed, these cells express glutamate receptors, which makes them vulnerable to glutamate through an increase in Ca^2+^ influx, production of ROS, and by induction of apoptosis. Moreover, OLs can be damaged indirectly by the activation of glutamate receptors in microglia, which leads to the release of proinflammatory cytokines [176].

Another mechanism responsible for white matter damage can be the activation of neuronal death pathways, which also occurs in epilepsy [177,178,179]. OLs need signals from neurons to survive. When neurons start to degenerate, the amount of survival factors significantly decreases, leading to OL injury and death [180,181]. Hence, OLs can be damaged secondary to neurons degeneration. 

### 3.4. Efficacy of MS and Epilepsy Modifying Drugs in Modulating Myelin Damage in the Context of MS and Epilepsy

Given the changes in white matter and their importance in the pathophysiology of epilepsy, therapeutics used in the treatment of MS may be effective against seizure-induced damages. Since neuroinflammation is a shared mechanism underlying MS and epilepsy pathogenesis, it is likely that MS-modifying drugs can preserve myelin and reduce epileptic activity. However, animal and clinical studies in this regard are still insufficient. Here, we summarize the present evidences of using MS-modifying drugs in epilepsy and discuss their effects on myelin content. 

Glatiramer acetate, an immunomodulatory medication, protected against hippocampal and cerebral cortex demyelination and decreased the frequency of epileptic seizures in PTZ induced epilepsy [182]. The therapeutic effects of fingolimod, the first oral medicine in the treatment of MS, which improves myelin repair and inflammation [183], have also been evaluated in the PTZ experimental model of epilepsy. Specifically, Gol et al. indicated that fingolimod could increase myelination and decrease the frequency of seizures in the hippocampus, together with a reduction in neuronal death and activation of astrocytes and microglia [184]. These results are further supported in the lithium–pilocarpine model, in which a decrease in the frequency and duration of seizures, neuronal loss, number of activated microglia and astrocytes, as well as in the decreased levels of IL-1β and TNFα in the hippocampus, are observed following fingolimod treatment [185]. The antiepileptic effects of fingolimod and its mechanisms were recently reviewed in details by Paudel [186]. Inhibition of Nogo receptor signaling enhances remyelination and functional restoration of myelin damage [187]. Lingo 1, a co-receptor of Nogo-66 receptor, is a potent inhibitor of OPCs’ differentiation. Hence, anti-Lingo 1 antibodies can decrease cognitive impairment and promote myelination in late EAE [188]. Moreover, downregulation of the Lingo-1 gene is reported to improve myelination in the lithium–pilocarpine model [128]. In turn, Natalizumab, a monoclonal anti-alpha4 integrin antibody treating relapsing-remitting MS, could reduce partial and generalized seizures in a patient with MS [189]. 

Likewise, there is evidence demonstrating the effectiveness of antiepileptic drugs on MS progression. For instance, the administration of valproic acid (VPA) decreased EAE clinical symptoms, demyelination, inflammation, and infiltration of immune cells into the spinal cord [190]. In addition, VPA also increased the number of remyelinated axons in the lesion area in focal EAE [191]. These effects might be mediated by reducing the expression of proinflammatory cytokines and lymphocyte proliferation, increasing the number of regulatory T cells in lymph nodes [192], inducing apoptosis in activated T cells [190], as well as recruiting endogenous progenitors into the lesion area [191]. In turn, Phenytoin, a sodium channel blocker used as an antiepileptic drug, could reduce EAE clinical scores and axonal degeneration of the corticospinal tract and dorsal column [193]. Besides, it has exhibited neuroprotective effects through reducing the loss of retinal nerve fiber layer thickness and macular volume in patients with acute optic neuritis [194]. However, some antiepileptic drugs, such as carbamazepine, could worsen the severity of MS depending on the dosage [195,196]. Therefore, both the dosage and the patient’s disease stage should be considered prior to using antiepileptic drugs for MS treatment.

Neurosteroids such as allopregnanolone may also target common pathological pathways in MS and epilepsy. Specifically, allopregnanolone may act as a positive modulator of inhibitory currents mediated by γ-aminobutyric acid type A (GABA_A_) receptors in epilepsy [197] In this way, the work developed by Lévesque et al. (2020) evidenced an effect of allopregnanolone in modulating ictogenesis and the occurrence of pathological network activity. Moreover, allopregnanolone treatment delayed the onset of spontaneous seizures in animal models of mesial temporal lobe epilepsy [198]. Regarding MS, allopregnanolone has been related with myelination induction and myelin protein synthesis in both PNS and CNS through the nuclear receptor for progesterone [199]. Moreover, it has been shown that the administration of allopregnanolone in mice with autoimmune demyelination ameliorated neurobehavioral deficits and improved the neuropathology and inflammation in the CNS [200]. These findings indicate that allopregnanolone and perhaps other neurosteroid-like compounds might represent new therapeutic strategies for both epilepsy and MS. 

## 4. Conclusions

Although epileptic seizure has been thought to be a comorbidity of MS for more than 150 years, this subject has attracted more attention recently. Furthermore, evidence in recent years have clearly shown that myelin damage is a comorbidity for epilepsy. Increased attention to shared pathological mechanisms underlying demyelination and epilepsy will provide a better insight into the link between MS and epilepsy, and could result in a better management. However, significant progress in determining the common pathological mechanisms in detail is still expected. This could help to develop therapeutic agents for managing both conditions. Likewise, a deeper understanding of the shared pathological mechanisms suggests that the disease modifying drugs for one pathology may help to manage the other. We hope that the current knowledge on the pathological events reviewed in this article help to provide a greater insight for researchers working on this bidirectional connection. 

## 5. Future Perspective

Even though the association between MS and epilepsy has been long introduced, the understanding of the pathological processes underlying this bidirectional association remains in its early days. Given that cortical lesions and atrophy in progressive MS is widespread, one may expect a higher prevalence of seizures among patients with SP-MS. However, there are still controversies of which clinical characteristics of MS are associated with the occurrence of epileptic seizures. Moreover, it is unclear to what extent seizures can exacerbate the clinical course and long-term prognosis of MS. Therefore, several fundamental questions should be addressed by further studies. Where do seizures start? Can MS lesions be epileptic foci? Which types of neurons are primarily affected in patients with MS that subsequently initiate seizures? Are they within the demyelinated lesions? Can epilepsy increase the risk of transition to progressive form in patients with RR-MS?

Inflammation and BBB integrity loss play important roles in the pathogenesis of both MS and epilepsy. However, the following question emerges: could BBB breakdown, myelin damage, and neuroinflammation—in the context of epilepsy—trigger a peripheral autoimmune response? 

Further investigations are required to clarify the suitable therapeutic approach for the management of both diseases. Some antiepileptic drugs such as valproic acid have shown great potential in enhancing remyelination and better outcomes in focal EAE, although the proposed underlying mechanism was a different one [191]. Clearly, our knowledge about disease-modifying drugs is still not sufficient. Consequently, it still needs to be clarified which disease-modifying drugs are the best suited for preventing the development of epilepsy in patients with MS, and which MS-modifying drugs would be better in restoring myelin in patients suffering from epilepsy.

## Figures and Tables

**Table 1:** Characteristics of patients with seizure associated demyelinating disorders.

Demyelinating Disease		Epilepsy Prevalence	Clinical Manifestation	MRI Findings	Most Frequent Seizure Type	Electroencephalographic (EEG) Characteristics	Possible Pathophysiological Mechanism	Ref.
Multiple sclerosis		0.5–8.3% with an average of 2.3%	Earlier onset of MS symptoms Worse cognitive performance in patients with frequent seizures or status epilepticus	Cortical and juxtacortical lesions Extensive cortical inflammation lower brain volumes Temporal lobe damage: Hippocampus, lateral temporal lobe, cingulate, and insula Cortical thinning and alteration of diffusion metrics in temporal lobe including insular cortex and cingulate gyrus	Partial secondary generalized	Diffuse asynchronous theta activity Synchronous rhythmic slow waves Focalized flattened EEG patterns Focal abnormalities	Temporal lobe cortical pathology Inhibitory GABA interneuron cell loss in layers IV and VI Reduced cortical thickness in the middle temporal gyrus Type I GMLs mostly in middle temporal gyrus Decreased GABA in left hippocampus and posterior cingulate cortex of RRMS Presence of cortical lesions Progressive brain atrophy	[25,26,27,28,42,43,44,45,46,47]
Progressive multifocal leukoencephalopathy		18%	New-onset seizures	Lesions adjacent to the hemispheric cortices	Simple and complex partial seizures Partial seizures with secondary generalization	-	-	[41]
Antibody-associated demyelination	MOG-IgG *	20.5% **	Encephalopathy Younger onset age Higher EDSS score Meningeal irritation Fever, headache, nausea and vomiting CSF leukocytosis	Inflammatory cortical brain lesions Subcortical white matter lesions Deep white matter lesion including periventricular and corpus callosum Cerebral peduncle less optic nerve and spinal cord involvement	Generalized tonic clonic seizure	Background theta to delta rhythm Intermittent low amplitude fast waves Focal sharp-wave Complex and asymmetric focal slow waves	-	[33,40,48,49]
	AQP4-IgG ***	1%	-	-	-	-	Slow K^+^ clearance	[33,50]

GABA: Gamma-Aminobutyric Acid; GMLs: Gray matter lesions; EDSS: Expanded Disability Status Scale; CSF: Cerebrospinal fluid; RRMS: relapsing-remitting MS. * MOG-Abs associated demyelination is related to several demyelinated disorders such as acute disseminated encephalomyelitis (ADEM), pediatric MS, transverse myelitis, optic neuritis (ON), and AQP4-Abs negative neuromyelitis optica spectrum disorders (NMOSD). ** A recent meta-analysis of 14 studies reported that general probability of seizure occurrence in patients with MOG-Ab-associated disease is 20.5%, particularly in children. Furthermore, the occurrence of seizure in patients with ADEM-like phenotype of MOG-Ab-associated disease is 37.3% [49]. *** Antibodies against AQP4 is a criterion for NMOSD diagnosis.

**Table 2:** Summary of published studies on epileptic seizures in patients with MS.

Type of Study	Number of Patient with MS	Patients with Seizures (Percentage)	Predominant Seizure Type					Seizure Occurrence at MS Onset	Seizure Occurrence before MS Onset	Seizure Occurrence after MS Onset	Ref.
			Simple Partial	Complex Partial	Secondary Generalized (sGTCS)	Generalized Tonic Clonic	Status Epilepticus				
Cohort	5041	102 (2%) In 67 patients (1.3%), epileptic seizure could not be explained by any cause other than MS	34 (50.7%)	Less frequent	28 (41.8%)	33 (49.3%)	18 (26.9%)	7 cases	26 case	69 case	[28]
Retrospective review of the records	310	10 (3.2%)	2	1 case of simple partial	6	2	2 cases of sGTCS	4 cases	Not reported	Not reported	[26]
Retrospective registered-based study	14,545	Cumulative incidence: 502 (3.5%)(CI 3.17–3.76) The 5-year prevalence: 1.7% (CI 1.54–1.98)	Single seizure was present in 3.0% (CI 2.77–3.32) of patients with MS				0.48%	Not reported	Not reported	Not reported	[52]
Systematic review	32 studies	Incidence: 2.28% (CI: 1.11–3.44%), at the range of 0.65–5.97% Prevalence: 3.09% (CI: 2.01–4.16%) at the range of 0.89–8.06%	Not reported	Not reported	Not reported	Not reported	Not reported	Not reported	Not reported	Not reported	[29]
Retrospective review of the records	1267 *	22 (1.74%)	Focal onset in 17 patients (77.3%)		14 out of 17 patients with MS (82.4%)	5 (22.7%)	3 (13%)	-	2 (9.1%)	16 (72.7%)	[51]
Cohort	428	13 (3%)	10 (77%)		Half of patient with focal seizure (38.5%)	3 (23%)	0	4 (31%)	-	8 **	[44]
Retrospective cross-sectional epidemiological study	431	19 (4.4%) 14 cases with active epilepsy	4 cases	3 cases	11 cases	-	5 (36%)	2	1	11 cases	[56]

* The first appearance of seizures for four patients (18.2%) could not be explained by the assessment of their medical records. ** One remaining patient was considered as clinically isolated syndrome. CI: 95% confidence interval; sGTCS: Secondary generalized tonic clonic seizure.

## Data Availability

Data sharing not applicable.
